# Anthelmintic resistance in small ruminants in the Nordic-Baltic region

**DOI:** 10.1186/s13028-021-00583-1

**Published:** 2021-04-27

**Authors:** Agnė Beleckė, Tomas Kupčinskas, Inga Stadalienė, Johan Höglund, Stig Milan Thamsborg, Snorre Stuen, Saulius Petkevičius

**Affiliations:** 1 Department of Veterinary Pathobiology, Faculty of Veterinary Medicine, Laboratory of Parasitology, Lithuanian University of Health Sciences, Tilžės 18, 47181 Kaunas, Lithuania; 2 Department of Biomedical Sciences and Veterinary Public Health, Swedish University of Agricultural Sciences, Box 7028, 750 07 Uppsala, Sweden; 3 Department of Veterinary and Animal Sciences, Section for Parasitology and Aquatic Pathobiology, University of Copenhagen, Dyrlægevej 100, 1870 Frederiksberg C, Denmark; 4 Department of Production Animal Clinical Sciences, Norwegian University of Life Sciences, Svebastadveien 112, 4325 Sandnes, Norway

**Keywords:** Gastrointestinal nematodes, Goat, Northern Europe, Sheep

## Abstract

Gastrointestinal nematodes (GIN) in small ruminants result in production losses, and consequently economic losses, and are an animal welfare problem in most countries in the Nordic-Baltic region. Intensive use of anthelmintics to control helminth infections has led to anthelmintic resistance (AR), which has become a major issue in many European countries. Several studies have been performed in countries in the Nordic-Baltic region (e.g. Denmark, Sweden, Norway and Lithuania) showing increasing/emerging levels of AR. The aim of this paper is to provide an overview of the problem of AR on sheep and goat farms in the Nordic-Baltic region. This region has a limited number of registered anthelmintics. However, researchers in this area have discovered some surprising findings, such as ivermectin (IVM) resistance on a farm that had never used IVM. In Sweden there is evidence of macrocyclic lactone (ML)-resistant *Haemonchus contortus* being introduced with sheep imported from the Netherlands. As elsewhere in the world, the livestock trade appears to be contributing to the spread of AR in the region and isolated cases of multidrug-resistant cases have also been reported. This is surprising given that the frequency of treatments here is much lower than in other countries where sheep production is economically more important. The prevailing nematodes are *Haemonchus*, *Teledorsagia* and *Trichostrongylus*, while on some farms *Haemonchus* is dominant and clinical haemonchosis has increasingly been observed in recent decades. The reasons for this are unclear, but are probably related to this parasite’s propensity to rapidly develop drug resistance and a general lack of awareness of the problem, possibly in combination with global warming and the increased livestock trade within the EU. In addition, domestic interactions through contacts with wildlife ruminants, alpacas may also be a contributing factor for transmission of AR.

## Background

Gastrointestinal nematode (GIN) infections remain the most prevalent and important parasitic disease in grazing ruminants worldwide, affecting the welfare and health of animals and resulting in significant economic losses, especially in the sheep and goat industry [1–3]. Infections with GIN may lead to diarrhoea and digestive problems, anaemia and even animal death but most commonly, infections are subclinical and result in an overall decrease in productivity [4–6].

From the early 1960s broad-spectrum anthelmintics administered routinely have helped to control GIN, but intensive and incorrect use of anthelmintics, under-dosing and continuous treatments with the same anthelmintics have led to nematode populations resistant to anthelmintic drugs [1, 7–9]. Anthelmintic resistance (AR) is a heritable genetic trait in worms to resist a normally effective dose of the anthelmintic, which gradually becomes widespread among the hosts [10]. Most on-farm screening studies are based on the faecal egg count reduction test (FECRT), sometimes in combination with in vitro tests, e.g. the egg hatch test (EHT) and the micro-agar larval development test (MALDT), or molecular tests. On some farms, there is not just resistance to a single drug, but to multiple or even all available anthelmintics. To date, this has mainly been documented on sheep farms in the UK [11–13], Germany [14], Switzerland [15] and Belgium [16]. The occurrence of AR, including multidrug resistance, seems to be increasing in the Nordic-Baltic region [17] as well, and this will complicate the control of GIN in small ruminants in general. Although the efficacy of different anthelmintics is evaluated occasionally in some countries, it should be emphasized that in most Nordic-Baltic countries, randomised FECRT surveys have not recently been carried out. This situation is seen as dangerous because regular monitoring programmes for detecting the development and spread of AR are important in order to detect the problem in due time and thus be able to take steps to prevent the nationwide spread of resistant strains.

The aim of this paper is to provide an overview of the problem of AR on sheep and goat farms in the Nordic-Baltic region. This region has a limited number of registered anthelmintics. However, researchers in this area have discovered some surprising findings, such as ivermectin (IVM) resistance on a farm that had never used IVM [18]. In Sweden there is evidence of macrocyclic lactone (ML)-resistant *Haemonchus contortus* being introduced with sheep imported from the Netherlands [19].

## Search strategy

This overview is based on a search in PubMed (http://www.ncbi.nlm.nih.gov/pubmed) using the terms “anthelmintic resistance or multidrug resistance, nematodes, goat, sheep, small ruminants”. The titles and abstracts were evaluated, and articles referring to AR were obtained and assessed in detail. The authors’ extensive experience with AR in small ruminants was used to critically evaluate the literature. Nematodes will be addressed at genus-level unless worm counts with proper identification of species have been performed.

## Review

Intensive use of anthelmintics will inevitably lead to AR in GIN. There is widespread emergence of nematodes resistant to benzimidazoles (BZ), levamisole (LEV) and IVM and other ML around the world and it is expanding in many parts of Europe. After a brief overview of AR of GIN in the EU, a more detailed account of AR in the Nordic-Baltic region is given below.

## Current AR situation in the EU

AR in parasites is now widespread throughout Europe and has increased over a 41-year period, although there are still gaps in knowledge in some regions, especially eastern Europe [20]. At the European level, resistance has developed not only in *Haemonchus,* but in *Teladorsagia, Trichostrongylus, Cooperia* and *Nematodirus* as well [20, 21]. In most countries, recent reports of AR mainly refer to cases of BZ resistance, with increasing numbers of cases of ML resistance, particularly to IVM [20, 22]. According to Vineer et al. [20], a meta-analysis of AR in sheep and goats in Europe since 2010 revealed average farm-level prevalences of resistance to BZ of 86%, to ML except moxidectin 52%, to LEV 48% and to moxidectin (MOX) 21%, excluding single-farm case reports. There have also been some reports of AR to doramectin in the Netherlands and to MOX in Switzerland and southern Germany [23, 24], as well as to monepantel (MOP) in the United Kingdom (UK) [25]. Multiple AR in sheep GIN has been detected in France, Greece and Italy [26]. Multidrug resistance has also been reported in the UK and Ireland and is an emerging problem in Scotland [12, 13, 27] and France [28].

The most widely used anthelmintics in small ruminants in Europe are BZ and IVM. AR to BZ has been found on up to 98% of sheep farms examined in the Czech Republic [29], 14% in Spain [30], 100% in the Slovak Republic [31] and 100% in Ireland [32]. In the UK, AR to BZ is 98% and in Scotland alone it is 64%. Furthermore, IVM and LEV are becoming ineffective in most parts of the UK [11–13, 25]. In Italy, AR and suspected resistance to BZ has been found on 40% of dairy goat farms and resistance to eprinomectin on 20% of these farms [33]. Meanwhile, in Belgium, AR to BZ, ML, closantel and MOP has been found on sheep farms [16] and in Poland, AR to BZ and to IVM has been detected on 89% and 76% of sheep farms respectively [34, 35].

## AR in the Nordic-Baltic region

AR is an increasing problem in all Nordic countries and several studies on this have been carried out in Denmark, Sweden, Norway and Lithuania. The first study on resistance in sheep nematodes carried out anywhere in the region was undertaken in Denmark and published in 1991, and described resistance to LEV in *Teladorsagia* [36]. Later, Maingi et al. (1996) reported evidence of BZ, IVM and LEV resistance in trichostrongyle nematodes in Danish goat herds, and the occurrence of AR to the BZ drug thiabendazole (4/13 herds), LEV (7/11 herds) and IVM (4/7 herds) on Danish sheep farms using FECRT and EHT [18, 37]. A 2014 study indicated that AR to the most commonly used anthelmintics was widespread in goat herds throughout Denmark. *Haemonchus contortus* was the predominant species in around half of the farms pre-treatment. Resistance to one or more anthelmintics was detected in nine out of 10 herds (90%). In the FECRT, resistance to BZ (fenbendazole) was present in all three herds tested, and to IVM in eight herds (80%). Based on EHT, resistance to BZ (EC50 value > 0.1 µg/ml) was found in 8 of the 10 herds [38]. Peña-Espinoza et al. (2014) also described a case of AR to BZ and IVM on one of the largest organic small ruminant farms in Denmark, whereas MOX and LEV were highly effective in the FECRT. BZ resistance in *H. contortus* and *T. colubriformis* was confirmed by the controlled efficacy test and pyrosequencing. IVM eliminated 100% of *H. contortus* and reduced *T. colubriformis* worm counts by 84–92% [39].

In Sweden, AR in small ruminants used to be relatively low. In a survey on sheep farms carried out in the 1990s, BZ (febantel and fenbendazole) was implicated in resistance in *H. contortus*, whereas IVM was highly effective [40]. Also, in a more recent study, only 4% (2/45) of randomly selected sheep farms showed evidence of BZ (albendazole) resistance in *H. contortus* by FECRT [41]. However, the allele frequency of the position of the codon 200 TAC mutation in the β-tubulin gene was measured by pyrosequencing and showed that BZ resistance was probably more widespread than that indicated by the FECRT. Parasitological diagnosis of “clinical” resistance to IVM was also found in two flocks. However, both the pre-treatment faecal egg counts and the reduction in these were low, and only three lambs had between 100 and 450 eggs/g faeces after treatment [41]. However, there has been evidence recently of emerging IVM resistance, especially in *H. contortus,* in Swedish sheep flocks. This is possibly due in part to the introduction of resistant parasites with animal imports from Europe, with resistance to IVM reported in a more recent case study on a limited number of farms but focusing on a farm on Gotland in the Baltic Sea that had imported sheep from the Netherlands [19]. Ongoing studies show that the situation has worsened recently, with more cases on mainland farms as well. On some farms, there are now strains that are multi-resistant to both BZ (albendazole) and IVM. There has also recently been a single case of triple-resistance including MOP, which appeared after just a few years of usage. Thus, on some farms the situation is alarming since the only available drug still showing efficacy is levamisole. Unfortunately there is a lack of information about the situation in goats [42].

A study in Norway based on FECRT showed that in randomly selected flocks of sheep, AR to BZ is presently at a low level (11%). However, when restricting the area to Rogaland County, eight out of ten (80%) sheep flocks, selected based on frequent deworming, application of the dose and move strategy or intensive use of grazing pasture, showed BZ resistance [43]. Recently, a case of AR to IVM has been detected in a flock of sheep in Norway [44]. AR to BZ has been detected in just one herd of goats, but only a limited number of animals have so far been tested [43].

Different surveys were conducted during 2013–2014 on Lithuanian sheep farms to detect AR [45, 46]. Based on FECRT, BZ resistance was found on three out of 15 farms (20%). Resistance to IVM was present on two out of 16 farms (13%) [45]. Based on in vitro studies, resistance to BZ was detected using two different methods. BZ-resistant GIN were found on all 25 farms investigated (100%) using EHT, and on 12 out of 17 farms (70.6%) using MALDT. AR to IVM using MALDT was detected on 13 out of 21 sheep farms (61.9%). AR to LEV using MALDT was detected on two out of six sheep farms (33%) [45, 46]. A survey to detect AR on goat farms in Lithuania was conducted in 2013. Using in vitro EHT, resistance to BZ (LD 50 > 0.1 µg/ml and LD 99 > 0.3 µg/ml) was detected on five out of seven goat farms (71%), AR to IVM was detected on all nine farms (100%), and AR to LEV was detected on two out of six farms (25%) [47].

No published studies on AR were found for other countries in the region.

## Anthelmintics and treatments in Nordic-Baltic region

Several questionnaire surveys to obtain information on small ruminant management and worm control practices in northern Europe have been undertaken.

One of the first surveys of sheep farmers was conducted in Denmark in 1993. Based on a questionnaire survey, the majority of farmers (97%) used anthelmintics. The mean number of treatments per year for lambs and adult sheep was 1.9 and 2.3 respectively. BZ was the most-used anthelmintic and 81% of farmers had been using the same class of anthelmintic for three or more years [37]. IVM had only been launched on the Danish market a few years earlier. Another publication in 2014 on Danish goat herds indicated that the mean number of treatments for both kids and adult goats was around one treatment per year. In 26% of the herds, kids and adults were treated with anthelmintics ≥ 2 times per year. Anthelmintic drugs were used in 89% of the herds, and 75% of these had a predetermined treatment plan, 63% used IVM as their last treatment, 17% used BZ, 8% used other drugs and 13% did not know which drug they had used [38].

A study in Norway from 2007 revealed that visual appraisal of individual weight was the most common means of estimating the anthelmintic dose used in flocks of sheep (79%) and herds of goats (85%). However, a recent study indicated that this method for estimating the actual dose in flocks of sheep has now reduced to 28% [48]. BZ was the predominant anthelmintic class used in sheep flocks (65% in 2007), whereas BZ and ML were used equally common in dairy goat herds. In the period 2005–2007, farmers of 46% of the sheep flocks never changed the anthelmintic class. The dose and move strategy was practised in 33% of the sheep flocks [49].

An online survey was conducted on 20 conventional and 19 organic commercial sheep farms in Sweden with more than 70 ewes. Only minor differences were found between organic and conventional producers. The grazing management routines were similar, with the majority (59%) having a combination of natural pastures and arable land grazed by animal groups moved between paddocks at least three times per season (67%). The parasite control methods were also similar, with the majority (97%) relying on anthelmintics. Both ewes and lambs were usually treated one to two times per year, often at different times of the year. The most-used anthelmintic was IVM, followed by BZ (albendazole). Treatment decisions were generally based on faecal egg counts, but some farmers relied on blanket treatments. Several farms (64%) reported alternating between different drug classes at least once a year or at every deworming event. Most farms (59%) had tested whether the anthelmintics used had the expected efficacy on at least one occasion [50].

In 2015–2016, 71 sheep farmers and 37 goat farmers were surveyed in Lithuania. Based on the results from the questionnaires, chemotherapy and/or chemoprophylaxis have been used by 72% of sheep farmers and 54% of goat farmers. In Lithuania, the most-used classes of anthelmintics were ML (only IVM was used) (67% and 65%) and BZ (28% and 20%) for sheep and goat treatments respectively. LEV was used very sporadically (4% and 15%) on sheep and goat farms respectively. Sheep were usually treated twice (63%) in spring before turnout and in autumn before housing, and goats were usually treated once (41%) after the start of the housing period between November and December. Yearlings and adults were usually treated together [47, 51]. No data are available for other countries in the region.

## Multidrug resistance and anthelmintic-resistant parasite genera

The evolution of multidrug resistance may lead to total anthelmintic failure [52]. Multidrug resistance is of growing concern in Nordic countries. According to data regularly collected by veterinarians for the Farm and Animal Health database in Sweden, the number of farms experiencing drug failure on occasion to both IVM and BZ is increasing, and in one case resistance to MOP as well [17]. Multidrug resistance has been detected in sheep farms in Lithuania. Using FECRT, multidrug resistance to BZ and ML was detected on a single farm. However, using in vitro tests, multidrug resistance to BZ and ML was detected on five out of 13 sheep farms (38.5%) [51]. In Denmark, using FECRT, multiple resistance to both IVM and BZ was found in seven goat herds (70%) [38]. Another study revealed an isolate of *T. colubriformis* to be resistant to both BZ and IVM [41]. Dual resistance to both IVM and BZ, based on FECRT, has also recently been recorded in single sheep flock in Norway [44]. There is a definite need for more studies on multidrug resistance in the region based on a randomised design.

In Norwegian goat herds, the pre-treatment infection levels of GIN were low compared with those in sheep flocks, and AR to BZ in *Teladorsagia* and *Trichostrongylus* was found in one goat herd [43]. In Lithuania, using different AR detection methods, *Teladorsagia* and *Trichostrongylus* were the dominant genera [45, 51]. In contrast, it appears that resistance is mainly associated with *Haemonchus* in Sweden, but other genera such as *Teledorsagia* and *Trichostrongylus* can also be involved according to droplet digital polymerase chain reaction data [53].

Based on a nematobiome study in Sweden, identified nematode species responded differently to IVM, BZ (albendazole) and LEV. Of practical interest is that both IVM and ABZ were unable to control *H. contortus* on several farms, whereas LEV still had reasonable efficacy. However, despite always having a high efficacy of treatment with LEV, *H. contortus* in particular survived at a low level. Similarly, *T. circumcincta* survived treatment with either IVM or BZ, but to a lesser extent than *H. contortus*, and not at all after LEV treatment [54]. In Denmark, AR was confirmed by the controlled efficacy test (CET) in *H. contortus* and *T. colubriformis* isolated from a farm with both sheep and goats [39].

## Conclusions

In sheep and goats in the Nordic-Baltic region, AR is common observed to IVM, BZ and LEV, which are the most commonly used anthelmintics in these countries. However, MOX and LEV are highly effective in northern Europe compared with the rest of Europe. Little is known about closantel and the relatively new drug MOP because these drugs are not applied or not registered in some countries, for example in Lithuania. As elsewhere in the world, the livestock trade appears to be contributing to the spread of AR in the region and isolated cases of multidrug-resistant cases have also been reported. This is surprising given that the frequency of treatments here is much lower than in other countries where sheep production is economically more important.

As in many other regions, many studies are case reports and surveys often suffer from lack of random sampling of farms. Studies on farms have been based on FECRT, MALDT, EHT and molecular methods. Although the gold standard for AR detection is CET, it is not a commonly used method in northern Europe because studies are usually conducted on commercial farms. In brief, standard methods for AR detection are needed not only in northern Europe but worldwide, and more thorough nationwide monitoring should be instituted [20].

In northern Europe the prevailing nematodes in sheep and goats are the same as in the rest of Europe: *Haemonchus*, *Teledorsagia* and *Trichostrongylus.* On some farms, *Haemonchus* is dominant and clinical haemonchosis has increasingly been observed in recent decades. The reasons for this are unclear, but are probably related to this parasite’s propensity to rapidly develop drug resistance and a general lack of awareness of the problem, possibly in combination with global warming and the increased livestock trade within the EU. The predicted patterns of climate change could alter the epidemiology of *Haemonchus* (and other strongyles) in northern Europe [55], and one obvious approach would be to undertake predictive mapping in order to be proactive in relation to impending changes in the climate that will affect this region. However, there are still some AR and GIN epidemiology data gaps in northern Europe and the Baltics, as for example no studies of this kind are available for Estonia, Latvia or Finland.

The key factors in slowing down the development of AR are now considered to be maintaining part of a parasite population in *refugia* (i.e. unexposed to drug, e.g. on pasture or in untreated animals), adopting novel non-chemical approaches, and improving pasture management and husbandry practices, and these should be included in any potential prophylactic control regimes suggested for GIN [21, 56]. However, there is little information about what level of *refugia* is required to avoid selection for resistance [57].

Improved diagnostics in wildlife ruminants, alpacas and imported small ruminants will help control the spread of AR in this region. Wild ruminants act as reservoirs for some GIN species (*Haemonchus*, *Cooperia*, *Oesophagostomum* and *Trichostrongylus*) that carry AR mutations as well as susceptible genotypes, and are a potential risk factor for transmission of AR [58–60]. This is certainly something that should be investigated in Nordic-Baltic conditions with the region’s large populations of cervids and their transboundary migrations. In addition, domestic interactions through contacts with “exotic” livestock, such as alpacas, may also be a contributing factor. It has been documented in some countries that imported camelids are sometimes infected with *Haemonchus* that are refractory to anthelmintic treatments [61, 62].

In conclusion, the present situation in regards to AR in the Nordic-Baltic region is not much different from what is observed in other European countries that have more intensive sheep and goat farming. However, a collaborative effort is required in the Nordic-Baltic region to exchange information on improved diagnostics, undertake mapping and draw up guidelines for the appropriate use of new drugs (monepantel, derquantel) or drugs that are being re-introduced, such as levamisol and closantel.

## Data Availability

The datasets used and/or analysed during the current study are available from the corresponding author upon reasonable request.

## Abbreviations

AR: Anthelmintic resistance
BZ: Benzimidazoles
CET: Controlled efficacy test
EC: Effective concentration
EHT: Egg hatch test
FECRT: Faecal egg count reduction test
GIN: Gastrointestinal nematodes
IVM: Ivermectin
LD: Lethal dose
LEV: Levamisole
MALDT: Micro-agar larval development test
ML: Macrocyclic lactones
MOP: Monepantel
MOX: Moxidectin

## Declarations

### Prior publication

Data have not previously been published.
